# First‐phase ejection fraction and ventricular‐arterial coupling across the lifespan: An in silico analysis

**DOI:** 10.14814/phy2.70855

**Published:** 2026-04-01

**Authors:** Edouard Long, Jose L. Flores‐Guerrero

**Affiliations:** ^1^ Faculty of Life Sciences and Medicine King’s College London London UK; ^2^ Institute of Cardiovascular Science University College London London UK; ^3^ MRC Unit for Lifelong Health and Ageing at UCL University College London London UK; ^4^ Interdisciplinary Center for Research and Science Education Autonomous University of Puebla Puebla Mexico

**Keywords:** ejection fraction, first‐phase ejection fraction, in silico, myocardial strain, systolic function, ventricular‐arterial coupling

## Abstract

First‐phase ejection fraction (EF1), the left ventricular ejection fraction (LVEF) up to maximal ventricular fiber shortening, has shown strong prognostic value in cardiovascular diseases, outperforming conventional LVEF, but remains uncharacterised in healthy individuals. An in silico cohort of healthy adults (*n* = 3837), from 25 to 75 years old was analyzed, showing good agreement with in vivo data. EF1 was derived from aortic flow waves as the proportion of end‐diastolic volume ejected up to peak aortic flow. Contractility (Ees) and afterload (Ea) were derived from pressure‐volume loops. Correlations were quantified using Pearson's coefficient and determinants were assessed using linear regression, adjusted for age and heart rate. Mean EF1 was 50 ± 3% versus LVEF 67 ± 2%, and EF1 was less influenced by age (*β*: 0.09 [95% confidence interval] [0.05, 0.12] vs. *β*: −0.27 [−0.30, −0.23]). EF1 correlated strongly with Ees compared to LVEF (*r*: 0.72 vs. 0.46, *p* < 0.001) and showed minimal correlation with Ea in contrast to LVEF (*r*: −0.10 vs. −0.37, *p* < 0.001). In regression, *β* was larger for Ees and Ea/Ees, and Ea smaller, in EF1 compared to LVEF. In conclusion, EF1 demonstrated a strong association with contractility and a negligible relationship with afterload, highlighting its potential as a sensitive, afterload‐independent measure of myocardial contractility.

## Graphical abstract

First‐phase ejection fraction (EF1), the left ventricular ejection fraction (LVEF) up to maximal fibre shortening, had a strong correlation with contractility and negligible relationship to afterload.

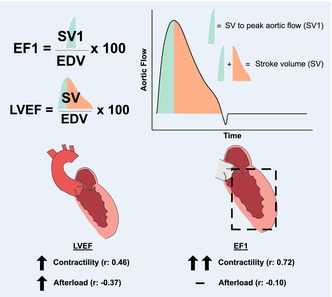

## 1 INTRODUCTION

First‐phase ejection fraction (EF1), the left ventricular ejection fraction (LVEF) up to the time of maximal fiber shortening, has been recently described as an index of myocardial contractility conceptualized on the basis that at the onset of heart failure (HF), the heart may be able to preserve total LVEF at the expense of slower sustained contraction (Gu et al., 2017).

The physiological mechanism responsible for this phenomenon has been linked to impaired shortening deactivation and mechanosensing‐based regulation of the myosin‐containing thick filament which enables “unlocking” of myosin motors in states of high stress in order to preserve total LVEF (Housmans et al., 1983; Lab et al., 1984; Linari et al., 2015; Reconditi et al., 2017). Consequently, dysregulation of these mechanisms may mask abnormalities in LV function before an overt drop in total LVEF.

The prognostic power of EF1 has been demonstrated in a range of cardiovascular diseases (CVDs) including aortic stenosis, heart failure, coronary artery disease, and pediatric CVD above and beyond more conventional measures such as LVEF or global longitudinal strain (GLS) (Gu et al., 2019; Gu, Singh, et al., 2021; Jin et al., 2024; Tang et al., 2025). However, as EF1 is a relatively novel concept, no reference values have been established and the current literature only describes EF1 in diseased individuals. Moreover, if EF1 is directly determined by a pathophysiological compensatory mechanism triggered by cardiomyocytes, it is possible that it may not vary with age in healthy adults. This is a key question that warrants investigation, as whether age‐adjusted thresholds for EF1 are required is unknown.

Thus, we sought to calculate reference values of EF1, investigate if it varies across the lifespan, and compare it with LVEF alongside ventricular‐arterial interaction via pressure‐volume loop (PVL) analysis.

## 2 METHODS

This was an observational cohort study performed on in silico data. Ethical approval for this study was not required as the dataset is freely available in the public domain and does not contain any personal identifiable data.

### 2.1 Dataset

The dataset used for this project was created by Charlton et al. to enable in silico evaluation of haemodynamic and pulse wave (PW) indexes (Charlton et al., 2019). It consists of virtual subjects, representative of healthy individuals, across the lifespan. The complete process by which the dataset was derived is described in the original publication. The plausibility of each virtual subject was determined by comparing simulated aortic and brachial mean arterial pressure (MAP), diastolic blood pressure (DBP), systolic blood pressure (SBP), pulse pressure (PP), and PP amplification (brachial to aortic PP ratio) to reference values from The Anglo‐Cardiff Collaborative Trial which included 4001 individuals aged 18–90 years (McEniery et al., 2005). Implausibility was defined as any value outside the 99% age‐specific confidence intervals (CI). Simulated PWs and derived indexes were compared to in vivo data and showed good agreement with well‐reproduced age‐related changes in haemodynamic parameters and PW morphology.

### 2.2 Derivation of left ventricular ejection fraction

As LVEF is not provided in the original dataset, it was mathematically derived from aortic root pressure waveforms using the method conceived by Swamy et al. which enables beat‐to‐beat estimation of LVEF from aortic pressure waves without requiring ventricular geometric assumptions (Swamy et al., 2009). A simplified overview of the method will be described, and technical details are provided in the Appendix A.

The method by Swamy et al. assumes a lumped parameter model of the cardiovascular system in which the LV is a chamber with a time‐varying elastance, the aortic valve is a one‐way opening, and the arterial system comprises a Windkessel model with two parameters: compliance and resistance. The derivation of LVEF is split into three steps. Firstly, the diastolic portion of the aortic pressure waveform is used to determine the arterial time constant. Next, using the arterial time constant, the systolic portion of the pressure waveform is linked to a mathematical model that describes how the LV's compliance changes during systole. Using the compliance and pressure data, relative volumes at the start and end of systole are then estimated. As LVEF is a ratio of these volumes, the unknown compliance cancels out, allowing calculation of an absolute LVEF value.

### 2.3 Derivation of first‐phase ejection fraction

**FIGURE 1:**
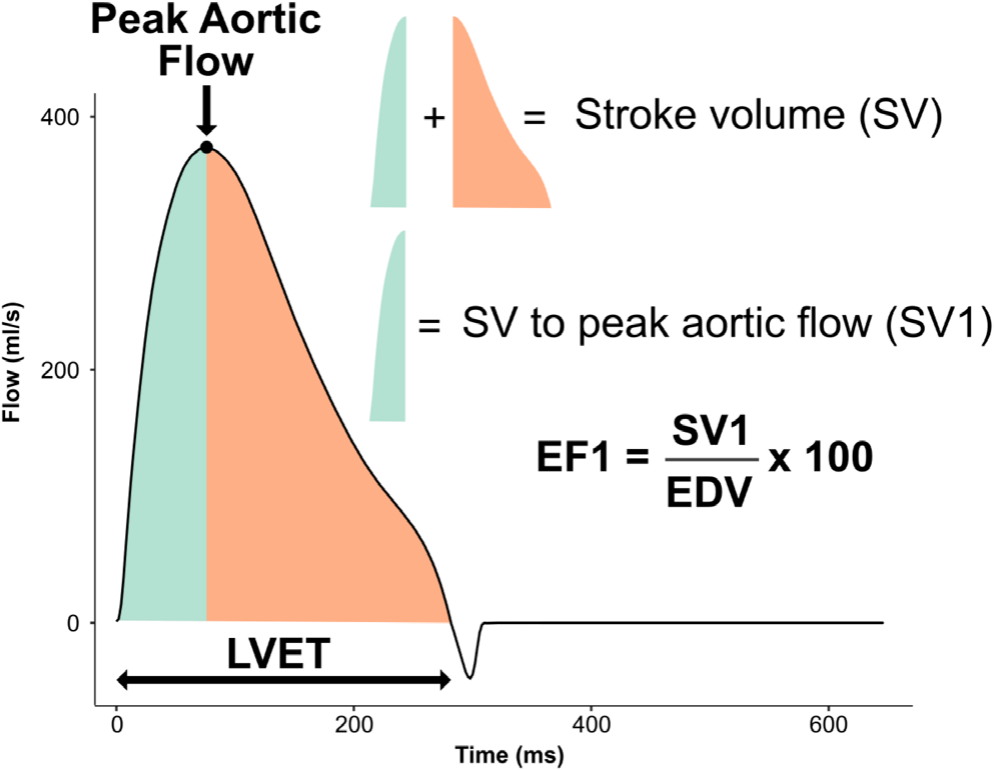
Derivation of first‐phase ejection fraction from an aortic flow wave. SV is equal to the area under the aortic flow curve (orange + green). The time of maximal ventricular fiber shortening is identified as peak aortic flow and the SV to peak aortic flow (SV1) is obtained using trapezoidal integration (green). EF1 can then be calculated as SV1/EDV × 100. EDV, end‐diastolic volume; EF1, first‐phase ejection fraction; SV, stroke volume; SV1, stroke volume to peak aortic flow.

EF1 was derived from aortic flow waves (Figure 1). Firstly, the time of maximal ventricular fiber shortening was assumed to be equal to the time of peak aortic flow in line with previously published methodology (Einarsen et al., 2021; Gu et al., 2019; Saeed et al., 2021). As the area under the aortic flow curve is proportional to total stroke volume (SV) (Charlton et al., 2019), the SV up to peak aortic flow (SV1) was derived using trapezoidal integration. This proportional estimate was scaled to the SV provided in the dataset to provide an absolute value of SV1. Subsequently, SV1 was divided by the end‐diastolic volume (EDV) (obtained during the derivation of LVEF) and multiplied by 100 to obtain EF1.

### 2.4 Derivation of ventricular‐arterial coupling indices

Quantification of ventricular‐arterial interaction was done in the pressure‐volume plane. End‐systolic pressure (ESP) was derived from aortic root pressure waveforms as the pressure at the dicrotic notch (Mehmel et al., 1981). Subsequently, effective arterial elastance (Ea) was calculated as ESP/SV and end‐systolic elastance (Ees) as ESP/end‐systolic volume (ESV). Ventricular‐arterial coupling was quantified as Ea/Ees (Suga & Sagawa, 1974).

### 2.5 Statistical analysis

Linear regression was used to assess the proportion of variance in EF1 and LVEF explained by age. All variables were standardized using *z*‐scores to enable comparison of coefficients between models. Assumptions were tested using Q‐Q plots to verify the normality of residuals, calculation of the variance inflation factor (VIF) to check for multicollinearity (with VIF >10 indicating a violation), influential values using Cook's distance, spread‐location plots to verify homoscedasticity, the Durbin‐Watson test for autocorrelation, and residuals‐fitted plots to test the linearity between individual predictors and the outcome of interest.

Subsequently, the correlations between EF1 and (1) Ees, (2) Ea, (3) Ea/Ees, and (4) LVEF were quantified with Pearson's correlation coefficient (*r*). To test for significant differences between the correlations of variables with LVEF and EF1, the Dunn and Clark method was used, which accounts for the fact that correlations are calculated from the same sample (Dunn & Clark, 1969). Multivariable linear regression was used to determine the proportion of EF1 and LVEF determined by (1) Ea, (2) Ees, and (3) Ea/Ees. Individual models, for both LVEF and EF1, were built for each of the three aforementioned variables and adjusted for age and heart rate (HR), which are known determinants of ventricular‐arterial coupling (Chantler et al., 2008). Standardization was performed to enable comparison of coefficients between models. All assumptions were tested as described in the previous paragraph.

All analyses were performed in R version 4.4.0 (R Foundation for Statistical Computing, Vienna, Austria). Statistical significance was defined as *p* < 0.05 for all statistical tests. Unless otherwise stated, data are presented as mean ± standard deviation (SD).

## 3 RESULTS

The haemodynamic characteristics of the study population (*n* = 3837) are displayed in Table 1. Individuals were aged 25 years old (18.7%), 35 years old (17.8%), 45 years old (17.0%), 55 years old (16.7%), 65 years old (15.3%), and 75 years old (14.5%). In terms of blood pressures, aortic SBP and brachial SBP broadly increased across the lifespan, whilst aortic DBP and brachial DBP decreased across the lifespan. Aortic MAP remained fairly constant. HR, left ventricular ejection time, and time to peak aortic flow showed little change across the lifespan.

**TABLE 1:** Haemodynamic characteristics.

Variable	Total (*N* = 3837)	25 years (*N* = 712)	35 years (*N* = 684)	45 years (*N* = 654)	55 years (*N* = 641)	65 years (*N* = 588)	75 years (*N* = 558)
Aortic SBP (mmHg)	109 ± 10	100 ± 8	105 ± 8	110 ± 8	112 ± 9	114 ± 9	115 ± 9
Aortic DBP (mmHg)	76 ± 7	75 ± 6	77 ± 6	79 ± 6	77 ± 6	75 ± 7	72 ± 7
Aortic MAP (mmHg)	94 ± 7	89 ± 6	93 ± 6	96 ± 6	96 ± 6	95 ± 6	94 ± 6
Brachial SBP (mmHg)	118 ± 9	112 ± 9	116 ± 9	120 ± 9	121 ± 9	120 ± 8	120 ± 9
Brachial DBP (mmHg)	73 ± 7	72 ± 6	74 ± 6	76 ± 6	75 ± 6	72 ± 7	69 ± 7
HR (bpm)	76 ± 9	73 ± 9	76 ± 9	77 ± 9	77 ± 9	76 ± 9	74 ± 9
LVET (ms)	283 ± 23	283 ± 23	284 ± 23	283 ± 23	282 ± 23	282 ± 23	282 ± 23
PFT (ms)	80 ± 0.1	80 ± 0.4	80 ± 0.0	80 ± 0.0	80 ± 0.0	80 ± 0.1	80 ± 0.2

*Note*: Values presented as mean ± SD. Data summarized as in Charlton et al. (2019).

Abbreviations: DBP, diastolic blood pressure; HR, heart rate; LVET, left ventricular ejection time; MAP, mean arterial pressure; PFT, time to peak aortic flow; SBP, systolic blood pressure; SD, standard deviation; SV, stroke volume.

### 3.1 Derivation of LVEF and EF1

Calculated ventricular volumes (EDV and ESV), SV, and SV1 derived from aortic flow and pressure waves are presented in Table 2. Both EDV and ESV decreased across the lifespan, with EDV showing a relatively greater decrease from 100 ± 21 mL in 25‐year‐olds to 81 ± 16 mL in 75‐year‐olds, compared to ESV which was 33 ± 8 mL in 25‐year‐olds and 28 ± 7 mL in 75‐year‐olds. SV1 also decreased with increasing age, going from 49 ± 8 mL in 25‐year‐olds to 41 ± 6 mL in 75‐year‐olds, although this was relatively smaller compared to the age‐related decrease in SV which went from 67 ± 13 mL in 25‐year‐olds to 54 ± 10 mL in 75‐year‐olds.

**TABLE 2:** Ventricular volumes across the lifespan.

Variable	Total (*N* = 3837)	25y (*N* = 712)	35 years (*N* = 684)	45 years (*N* = 654)	55 years (*N* = 641)	65 years (*N* = 588)	75 years (*N* = 558)
SV (mL)	60 ± 12	67 ± 13	64 ± 13	61 ± 12	59 ± 11	56 ± 10	54 ± 10
SV1 (mL)	45 ± 8	49 ± 8	47 ± 8	45 ± 7	44 ± 7	42 ± 7	41 ± 6
EDV (mL)	90 ± 19	100 ± 21	95 ± 20	91 ± 18	88 ± 18	84 ± 17	81 ± 16
ESV (mL)	30 ± 7	33 ± 8	31 ± 8	30 ± 7	29 ± 7	28 ± 6	28 ± 7

*Note*: Values presented as mean ± SD.

Abbreviations: EDV, end‐diastolic volume; ESV, end‐systolic volume; SD, standard deviation; SV, stroke volume; SV1, stroke volume up to peak aortic flow.

**FIGURE 2:**
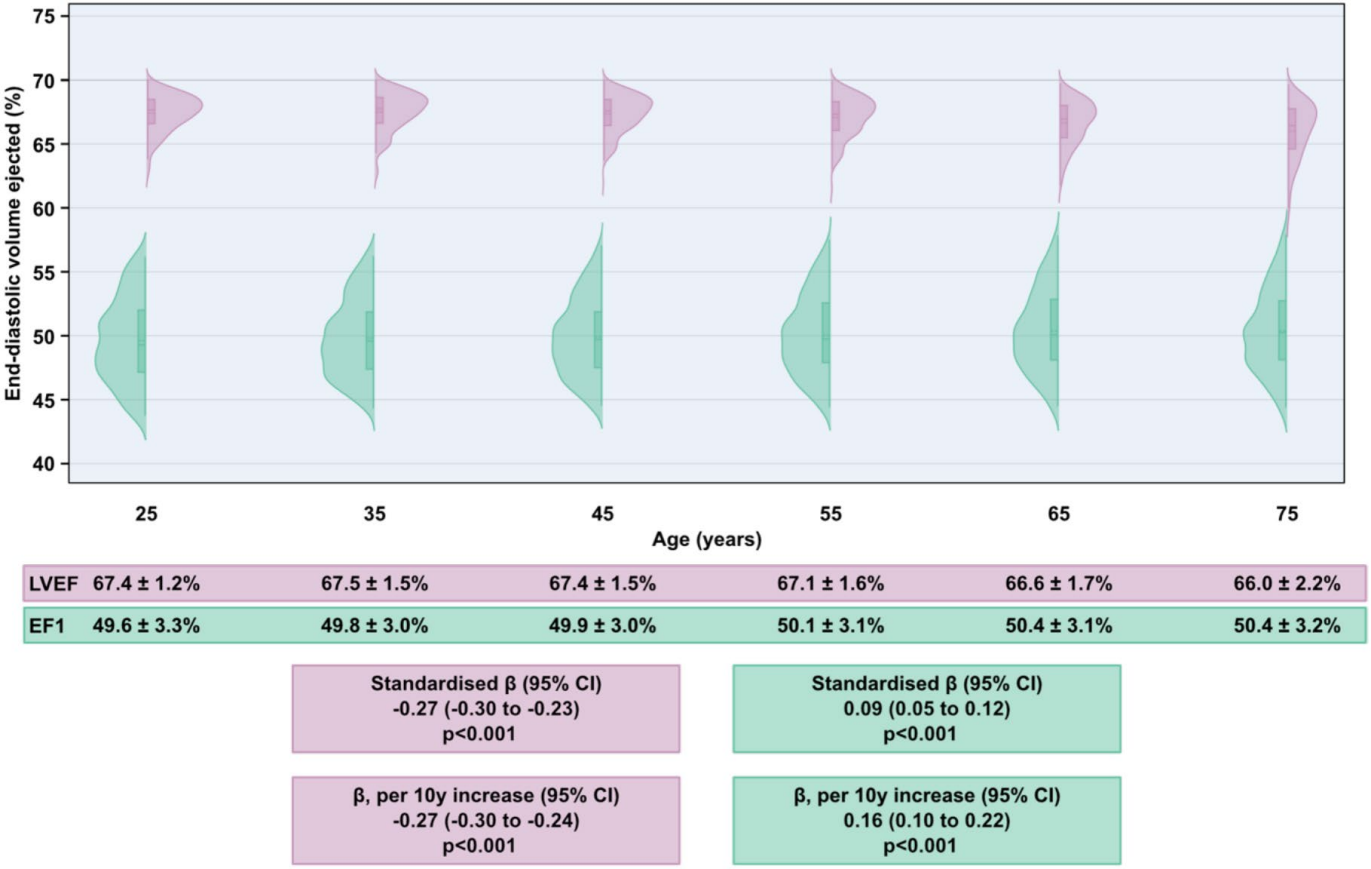
Distribution of EF1 and LVEF across the lifespan. EF1 displayed a wider distribution across decades but was less related to age than LVEF. Violin plots show the distribution of the variable, with internal box plots showing the median and interquartile range. Standardized *β* represents change in LVEF or EF1 (in SD) per SD increase in age. *β* per 10 years increase represents change in LVEF or EF1 (in %) per 10 years increase in age. CI, confidence interval; EF1, first‐phase ejection fraction; LVEF, left ventricular ejection fraction; SD, standard deviation.

**FIGURE 3:**
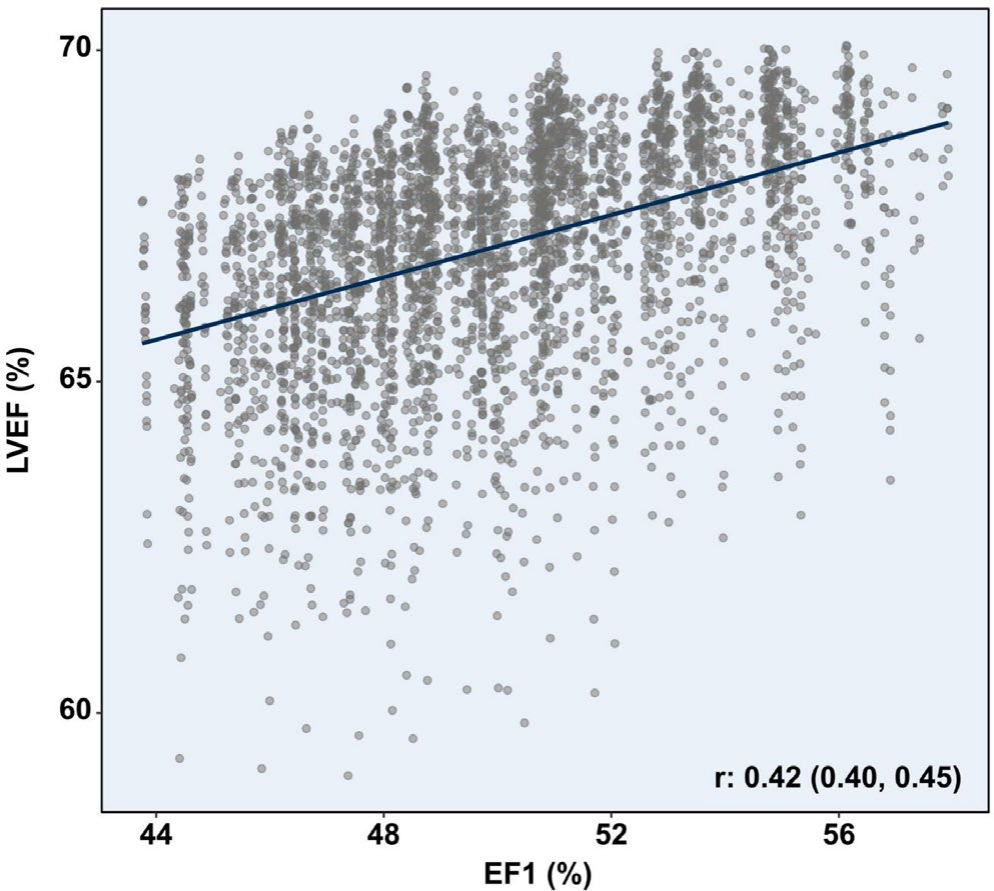
Correlation between EF1 and LVEF. EF1 had a modest correlation with LVEF. Pearson's correlation coefficient shown with corresponding 95% confidence intervals (*p* < 0.001). EF1, first‐phase ejection fraction; LVEF, left ventricular ejection fraction.

Subsequently, EF1 and LVEF were calculated (Figure 2). Broadly, EF1 (total: 50 ± 3%) showed a wider distribution of values but smaller change across the lifespan compared to LVEF (total: 67 ± 2%), which showed a relatively larger decrease across the lifespan. In linear regression analysis, age was less strongly associated with EF1 (*β*: 0.09, 95% CI: 0.05–0.12, *p* < 0.001) than with LVEF (*β*: −0.27, 95% CI: −0.30 to −0.23, *p* < 0.001). EF1 and LVEF had a modest correlation with each other (*r*: 0.42, 95% CI: 0.40–0.45) (Figure 3).

### 3.2 Relationship of EF1 and LVEF with indices of ventricular‐arterial interaction

**FIGURE 4:**
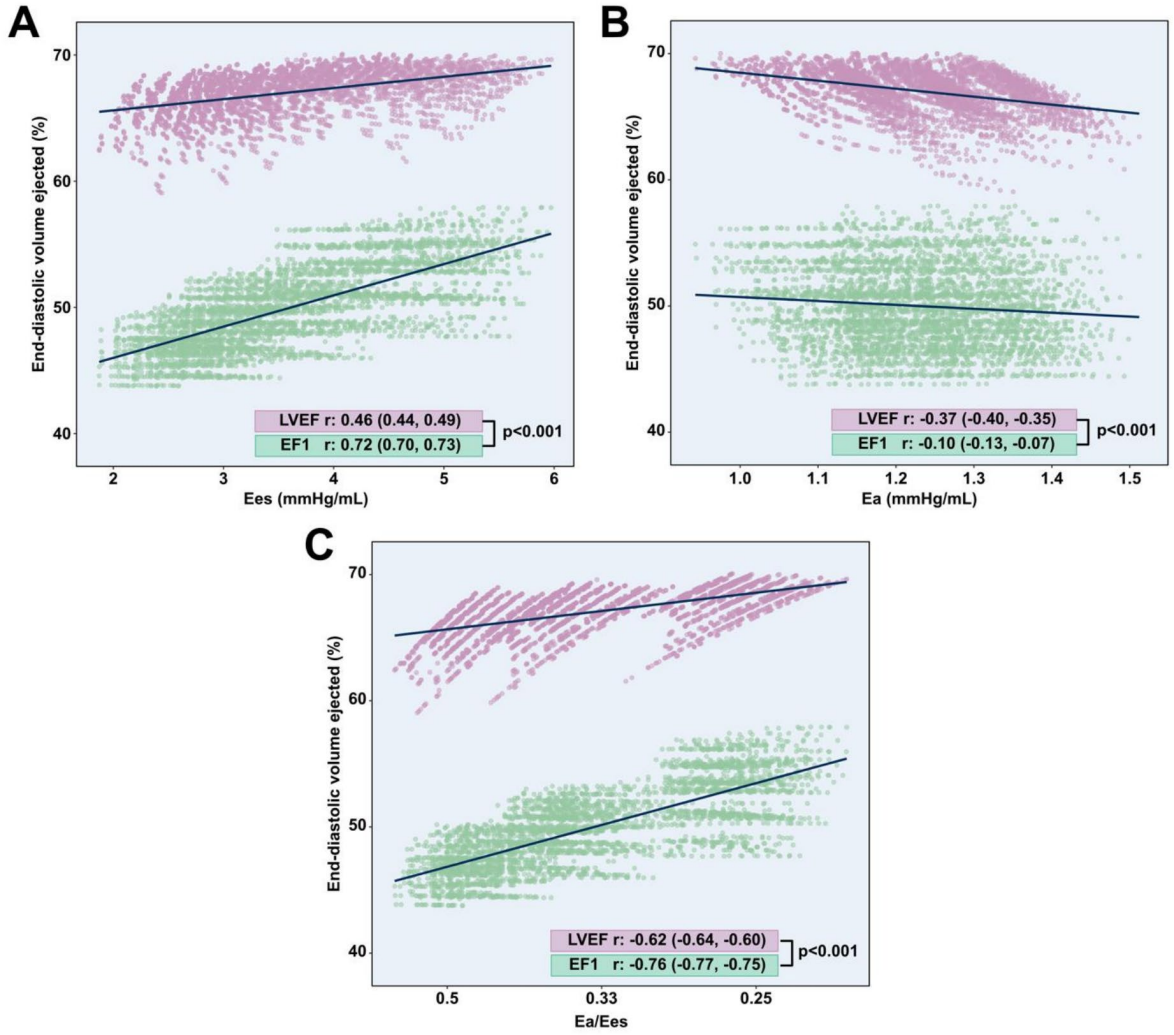
Correlations of EF1 and LVEF with Ees, Ea, and Ea/Ees. EF1 displayed significantly stronger correlations with Ees (A) and Ea/Ees (C), whereas LVEF displayed a stronger correlation with Ea (B). Clustering in C reflects the discrete parametrisation of Ea and Ees during simulation, which were sampled at stepwise intervals. Correlations quantified using Pearson's correlation coefficient (all *p* < 0.001) with respective 95% confidence intervals. LVEF and EF1 correlations compared using the Dunn and Clark method. Ea, effective arterial elastance; Ees, end‐systolic elastance; EF1, first‐phase ejection fraction; LVEF, left ventricular ejection fraction.

Individual correlations across the whole cohort between LVEF and EF1 with Ees, Ea, and Ea/Ees are shown in Figure 4. EF1 showed strong correlations with Ees (*r*: 0.72, 95% CI: 0.70–0.73) and Ea/Ees (*r*: −0.76, 95% CI: −0.77 to −0.75) and a negligible correlation with Ea (*r*: −0.10, 95% CI: −0.13 to −0.07). LVEF showed a good correlation with Ea/Ees (*r*: −0.62, 95% CI: −0.64 to −0.60) and a modest correlation with Ees (*r*: 0.46, 95% CI: 0.44 to 0.49) and Ea (*r*: −0.37, 95% CI: −0.40 to −0.35).

Comparing EF1 and LVEF, EF1 had a significantly stronger correlation with Ees (*p* < 0.001) and Ea/Ees (*p* < 0.001). LVEF had a significantly stronger correlation with Ea (*p* < 0.001).

### 3.3 Regression analysis

In multivariable regression assessing the predictors of LVEF and EF1 (Table 3), adjusted for age and HR, EF1 was more strongly associated with Ees (*β*: 0.78, 95% CI: 0.76–0.80, *p* < 0.001) compared to LVEF (*β*: 0.65, 95% CI: 0.64–0.68, *p* < 0.001), as indicated by its larger standardized *β*. Conversely, Ea had a stronger association with LVEF (*β*: −0.32, 95% CI: −0.35 to −0.29, *p* < 0.001) compared to EF1 (*β*: −0.19, 95% CI: −0.22 to −0.16, *p* < 0.001). Finally, Ea/Ees had a stronger association of EF1 (*β*: −0.76, 95% CI: −0.78 to −0.74, *p* < 0.001) compared to LVEF (*β*: −0.72, 95% CI: −0.74 to −0.70, *p* < 0.001). The same results were seen in unadjusted analysis.

**TABLE 3:** Association of contractility, afterload, and ventricular‐arterial coupling with LVEF and EF1.

Independent variable	Unadjusted		Adjusted for age and HR	
	Standardized *β* (95% CI)	*p*‐value	Standardized *β* (95% CI)	*p*‐value
Dependent variable: LVEF				
Ees (mmHg/mL)	0.46 (0.43, 0.49)	**<0.001**	0.65 (0.64, 0.68)	**<0.001**
Ea (mmHg/mL)	‐0.37 (−0.40, −0.35)	**<0.001**	‐0.32 (−0.35, −0.29)	**<0.001**
Ea/Ees	−0.62 (−0.65, −0.60)	**<0.001**	−0.72 (−0.74, −0.70)	**<0.001**
Dependent variable: EF1				
Ees (mmHg/mL)	0.72 (0.69, 0.74)	**<0.001**	0.78 (0.76, 0.80)	**<0.001**
Ea (mmHg/mL)	−0.10 (−0.13, −0.07)	**<0.001**	−0.19 (−0.22, −0.16)	**<0.001**
Ea/Ees	−0.76 (−0.78, −0.74)	**<0.001**	−0.76 (−0.78, −0.74)	**<0.001**

*Note*: *β* represents change in LVEF or EF1 (in SD) per SD increase in variable (Ea, Ees, Ea/Ees). Significant *p*‐values shown in bold.

Abbreviations: Ea, effective arterial elastance; Ees, end‐systolic elastance; EF1, first‐phase ejection fraction; LVEF, left ventricular ejection fraction; SD, standard deviation.

## 4 DISCUSSION

In the present study we have described, for the first time, reference values for EF1 and compared its determinants to LVEF. Our key findings can be summarized as follows: first, EF1 showed a wider distribution of values across the lifespan and was less influenced by age. Second, EF1 had a moderate correlation with LVEF. Third, EF1 had a strong correlation with contractility and ventricular‐arterial coupling, whereas its correlation with afterload was negligible. Finally, compared to LVEF, EF1 had a significantly stronger association with contractility and ventricular‐arterial coupling, and a significantly weaker association with afterload.

### 4.1 Reference values for EF1 across the lifespan in healthy adults

To the best of our knowledge, EF1 has not yet been assessed in healthy individuals and so it is difficult to compare the calculated values in this study. Reassuringly, values obtained for EDV and ESV are within reported reference ranges (19). Upon closer inspection, volumes may, on average, be smaller than reported in the literature. Nevertheless, as both LVEF and EF1 are proportions, the absolute value of volumes is of smaller concern provided the bias in their estimation is consistent. In a study investigating EF1 in patients with coronary artery disease by Minczykowski et al., a control group of 50 healthy volunteers had EF1 measured on echocardiography, and the median value was 37.0% (interquartile range: 30.0%–40.0%) (Minczykowski et al., 2023). This suggests that the method applied in this study, using aortic flow waves, may yield larger absolute values of EF1 compared to echocardiography. Inter‐modality differences in measurements are not uncommon and, in the context of EF1, have been found on cardiovascular magnetic resonance (CMR) imaging, with measurement on CMR yielding lower values of EF1 compared to echocardiography (mean difference: −5.0% ± 7.8%) (Gu, Bing, et al., 2021).

With respect to the relationship between age and EF1, the majority of published studies that assessed the determinants of EF1 found that age was not a significant predictor of EF1 (Bing et al., 2020; Carter‐Storch et al., 2021; Gu et al., 2017, 2024; Gu, Bing, et al., 2021; Gu, Singh, et al., 2021; Minczykowski et al., 2023), although these have generally included older adults with established CVD, which would limit their power to detect subtle age‐related differences. Although a significant relationship between EF1 and age was found in this study, the effect size was small (*β*: 0.09), which suggests that disease‐specific cut‐offs for EF1 may not require adjustment in patients of different ages. However, it should be noted that LVEF is known to vary by sex and race and so it would be important to investigate whether these may be potential determinants of EF1 (Pedrizzetti et al., 2025; Poppe et al., 2015).

A moderate correlation (*r*: 0.42, 95% CI: 0.40 to 0.45) was found between EF1 and LVEF within the study population, which is comparable to that reported by Carter‐Storch et al. (*r*: 0.32, *p* < 0.001) in a prospective cohort of 203 patients scheduled for aortic valve replacement (Carter‐Storch et al., 2021), although others have found conflicting results (Bing et al., 2020; Jin et al., 2024; Minczykowski et al., 2023). The small number of studies, alongside the heterogeneous patient cohorts, no doubt contributes to these discrepant results. Moreover, certain pathological states may alter the degree of agreement between EF1 and LVEF.

### 4.2 Relationship with ventricular contractility

EF1 exhibited a strong correlation with ventricular contractility, measured by Ees, and EF1's correlation with Ees was significantly stronger than LVEF's across the lifespan. In multivariable regression, Ees was also more associated with EF1 than LVEF.

As EF1 is a measure of peak systolic strain rate and LVEF of global systolic function, the results of this study are in line with evidence demonstrating peak systolic strain rate to better reflect changes in intrinsic myocardial contractility, compared to LVEF and regional systolic strain which are influenced by changes in loading conditions and HR (D'hooge, 2000; Weidemann et al., 2002). Furthermore, Einarsen et al. demonstrated EF1 to be closely related to mean peak systolic mitral annulus velocity (S′) on echocardiography, which itself has been closely related to LV contractility (Thorstensen et al., 2011). The correlation of LVEF with Ees seen in this study (*r*: 0.46) is comparable to that found by Monge García et al. using invasive pressure‐volume loop measurements in vivo (*r*: 0.43) (Monge García et al., 2019), strengthening the plausibility of our results.

### 4.3 Relationship with afterload

EF1 showed a negligible relationship with ventricular afterload (Ea). It has long been known that LVEF is relatively load dependent, which represents one of its limitations (Konstam & Abboud, 2017). This phenomenon is exemplified in septic patients, where a preserved LVEF may not indicate normal LV contractility, but rather reflect substantial reductions in afterload whereas an acute decline in LVEF following correction of arterial hypotension with vasopressors may not signify impaired LV contractility, but instead reveal pre‐existing LV dysfunction (Vieillard‐Baron et al., 2008). In the context of aortic stenosis, where EF1 has been studied extensively, it has been shown that reduced LVEF may be primarily related to high afterload as opposed to myocardial fibrosis (Rajah et al., 2025). On the other hand, CMR studies have shown that the impact of extracellular matrix expansion and replacement fibrosis, due to chronically increased afterload in aortic stenosis, can be detected by EF1 (Gu, Bing, et al., 2021). Therefore, this supports the hypothesis that EF1 is less sensitive to increased afterload and, in the context of aortic stenosis, is able to quantify myocardial contractility independently of afterload severity (Einarsen et al., 2021).

### 4.4 Relationship with ventricular‐arterial coupling

Ventricular‐arterial coupling, quantified by Ea/Ees, was found to be strongly related to EF1 and superior to LVEF. As Ea/Ees provides a comprehensive quantification of ventricular function and is known to be prognostic (Ikonomidis et al., 2019; Ky et al., 2013), it is promising that EF1 has a stronger correlation with Ea/Ees than LVEF and further suggests it may not only provide a more detailed haemodynamic assessment of cardiac function but also improve prognostication. The global correlation between LVEF and Ea/Ees in this study is also comparable to that obtained in vivo by Monge García et al., lending plausibility to these results. Nevertheless, it should be remembered that Ea/Ees has major limitations as an index of ventricular‐arterial interaction, namely its inability to characterize the LV loading sequence (Gu et al., 2017; Linari et al., 2015), and the ratio of pulse wave velocity (PWV) to GLS is also recommended as an alternative measure of ventricular‐arterial coupling. Therefore, the index of PWV/EF1 also warrants investigation as it may provide a robust measure of both myocardial contractility alongside pulsatile and resistive arterial load, although this remains to be demonstrated in future studies. Nevertheless, there remains a need to interrogate further parameters of ventricular‐arterial interaction, across a range of modalities, to fully characterize the determinants of EF1.

### 4.5 Limitations

Several limitations to this study should be noted. First, due to the in silico nature of this analysis, these results should be seen as hypothesis‐generating and require validation in real patient cohorts. Nevertheless, the PWs and their corresponding haemodynamic indices have been demonstrated to provide good agreement with corresponding in vivo data (Charlton et al., 2019). Second, although the noise‐free nature of in silico data is one of its strengths, it may also yield overly precise measurements and effect sizes, alongside seemingly predictable distributions due to clustering, which may not be seen with in vivo data which is inherently noisier. Moreover, the deterministic nature of in silico simulations yields minimal random variability, which may result in artificially strong correlation coefficients and narrow CIs compared to in vivo data where biological heterogeneity would introduce additional variance which may weaken observed associations. Third, although parameters were standardized to enable relative comparisons between models, this limits the generalisability of our findings in datasets with different distributions and ranges of variables. Fourth, in relation to the clinical variables used, it is known that LVEF differs by sex and ethnicity, which was not available in this dataset. However, in the context of EF1, most studies have found sex is not a significant confounder (Bing et al., 2020; Carter‐Storch et al., 2021; Feder et al., 2023; Gu et al., 2024; Gu, Bing, et al., 2021; Minczykowski et al., 2023). Fifth, the method used to derive LVEF relies on the assumption that the lumped parameter model is an accurate representation of the aortic pressure waveform, arterial compliance is constant, and the three‐parameter raised cosine function provides an adequate representation of LV elastance (Swamy et al., 2009). However, the accuracy of this method has been demonstrated in vivo, with a root‐mean‐squared error of 5.6% relative to standard echocardiography (Swamy et al., 2009). Sixth, in both the calculation of LVEF and Ees, the ventricular unstressed volume ($V_{0}$) was assumed to be 0 mL, as single‐beat estimation of $V_{0}$ requires ECG signal tracings, which were not available in this dataset. Physiologically, it is known that $V_{0}$ varies over time and is subject‐specific, meaning that our calculated Ees used as the reference evaluation for systolic function is potentially inaccurate (Sagawa, 1981). However, prior studies have also approximated $V_{0}$ as 0 mL and Heerdt et al. have demonstrated 100% concordance between experimentally measured and simulated contractility with $V_{0}$ fixed at 0 mL (Arvidsson et al., 2023; Heerdt et al., 2018). Finally, the index of Ea/Ees has inherent limitations as a measure of ventricular‐arterial interaction and so the findings of the present study should be interrogated with other measures of ventricular‐arterial interaction, particularly those concerning pulsatile arterial load (Ikonomidis et al., 2019).

## 5 CONCLUSIONS

The present study has described reference values for EF1 across the lifespan, alongside EF1's relationship with indices of ventricular‐arterial coupling and how this contrasts with LVEF. In healthy individuals, EF1 derived from aortic flow waves remained around 50% across the lifespan and was less influenced by age compared to LVEF. EF1 showed a strong correlation with ventricular contractility and a negligible relationship with afterload, demonstrating its potential role as a sensitive index of myocardial contractility that is unaffected by alterations to afterload, in contrast to LVEF. EF1 also had a stronger relationship with ventricular‐arterial coupling compared to LVEF.

## AUTHOR CONTRIBUTIONS

**Edouard Long:** Conceptualization; data curation; formal analysis; investigation; methodology; project administration; resources; software; supervision; validation; visualization. **Jose L. Flores‐Guerrero:** Conceptualization; investigation; methodology; supervision.

## FUNDING INFORMATION

The authors declare that no financial support was received for the writing and publication of this manuscript.

## CONFLICT OF INTEREST STATEMENT

The authors declare no conflicts of interest related to this work.

## ETHICS STATEMENT

Ethical approval for this study was not required as the dataset is freely available in the public domain and does not contain any personal identifiable data.

## Data Availability

The data underlying this article are available at https://doi.org/10.5281/zenodo.3374476.

## APPENDIX A

### A.1 LUMPED PARAMETER MODEL OF LVEF

The lumped parameter model representing the aortic pressure waveform is based on an electrical analogue where voltage = pressure (P), charge = volume (V), current = flow rate, the aortic valve is an ideal diode, the LV is a variable capacitor with compliance ($C_{\mathrm{LV}}$), whose elastance ($E_{\mathrm{LV}}=\frac{1}{C_{\mathrm{LV}}\;}$) oscillates over time (t) to drive the flow of blood, and the arteries (a) are a two‐parameter Windkessel model accounting for the resistance ($R_{a}$) of small arteries and compliance ($C_{a})$ of large arteries. Alongside compliance, the LV and large arteries are parametrised with an unstressed filling volume ($V_{0}$). Beat‐to‐beat, $C_{a}$ is assumed to be constant whereas other parameters are assumed to vary.

Subsequently, the pressure‐volume relationship of the LV during systole is defined as:

(1)
$$V_{\mathrm{LV}}\left(t\right)=\frac{P_{a}\left(t\right)}{E_{\mathrm{LV}}\left(t\right)}+V_{0},t_{\mathrm{bs}}<t\leq t_{\mathrm{es}}$$

where bs and es subscripts represent the beginning and end of the systolic ejection interval, respectively. The governing equation of the model is:

(2)
$$-\frac{d}{\mathrm{dt}}\;\frac{\;P_{a}\left(t\right)}{\;E_{\mathrm{LV}}\left(t\right)}=C_{a}\frac{d}{\mathrm{dt}}P_{a}\left(t\right)+\frac{\;P_{a}\left(t\right)}{\;R_{a}},t_{\mathrm{bs}}<t\leq t_{\mathrm{es}}$$

This equation is then integrated from $t_{\mathrm{bs}}$ to time t within the systolic ejection interval and divided by the constant scale factor $\;C_{a}$, where the Windkessel time constant $\tau =R_{a}\;C_{a}$:

(3)
$$\begin{array}{c} \frac{\;P_{a}\left(t_{\mathrm{bs}}\right)}{\;C_{a}\;E_{\mathrm{LV}}\left(t_{\mathrm{bs}}\right)}-\frac{\;P_{a}\left(t\right)}{\;C_{a}\;E_{\mathrm{LV}}\left(t\right)}\\ =P_{a}\left(t\right)-P_{a}\left(t_{\mathrm{bs}}\right)+\frac{1}{\tau }\int_{t_{\mathrm{bs}}}^{t}P_{a}\left(\lambda \right)\mathrm{d\lambda},t_{\mathrm{bs}}<t\leq t_{\mathrm{es}} \end{array}$$

To enable fitting of the model to an aortic pressure waveform, the equation is discretised by replacing t with nT, where n demotes discrete time and T is the sampling period. The integral is then approximated using the trapezoidal formula:

(4)
$$\begin{array}{c} \frac{\;P_{a}\left(n_{\mathrm{bs}}T\right)}{\;C_{a}\;E_{\mathrm{LV}}\left(n_{\mathrm{bs}}T\right)}-\frac{\;P_{a}\left(\mathrm{nT}\right)}{\;C_{a}\;E_{\mathrm{LV}}\left(\mathrm{nT}\right)}=P_{a}\left(\mathrm{nT}\right)-P_{a}\left(n_{\mathrm{bs}}T\right)\\ +\frac{T}{2\tau }\sum_{k=n_{\mathrm{bs}}+1}^{n}\left(\;P_{a}\left(\mathrm{kT}\right)+P_{a}\left(\left(k-1\right)T\right)\right),n_{\mathrm{bs}}<n\leq n_{\mathrm{es}} \end{array}$$

Finally, to arrive at a solvable set of equations, the temporal evolution of $\;E_{\mathrm{LV}}\left(t\right)$ over a cardiac cycle is assumed to be characterized using a parametric raised cosine function:

(5)
$$\;E_{\mathrm{LV}}\left(t\right)=\left\{\begin{array}{c} E_{\min}+\frac{E_{\max}-E_{\min}}{2}\left\{1-\cos\left(\frac{\pi \left(t-t_{\mathrm{bi}}\right)}{T_{s}}\right)\right\},t_{\mathrm{bi}}\leq t<t_{\mathrm{bi}}+T_{s}\;\\ E_{\min}+\frac{E_{\max}-E_{\min}}{2}\left\{1+\cos\left(\frac{2\mathrm{\pi t}\left(t-\left(t_{\mathrm{bi}}+T_{s}\right)\right)}{T_{s}}\right)\right\},t_{\mathrm{bi}}+T_{s}\leq t<t_{\mathrm{bi}}+1.5T_{s}\\ \;E_{\min},\;t_{\mathrm{bi}}+1.5T_{s}\leq t\; \end{array}\right\}$$

Substitution of the parametric raised cosine function into the discretized Equation (4), alongside the assumption that $C_{a}E_{\min}=0.05\times C_{a}E_{\max}$, leaves four unknowns: τ, $C_{a}E_{\max}$, $T_{s}$, and $C_{a}\;E_{\mathrm{LV}}\left(n_{\mathrm{bs}}T\right)$, which are estimated beat‐to‐beat in two steps.

Firstly, τ is estimated from the diastolic portion of the aortic pressure waveform by least squares fitting of an exponential to $\;P_{a}$ between the dicrotic notch of the waveform and the ensuing minimum pressure. Due to the in silico nature of the dataset, $P_{a}\left(t\right)$ was assumed to be noise‐free and so τ was estimated from a single cardiac cycle. In the second step, the calculated value of τ is substituted into Equation 4 and $C_{a}E_{\max}$, $T_{s}$, and $C_{a}\;E_{\mathrm{LV}}\left(n_{\mathrm{bs}}T\right)$ are estimated using least squares optimisation of the equation to the systolic ejection interval of the aortic pressure waveform.

Beat‐to‐beat LVEF can then be calculated from the fitted parameters, with $V_{0}$ assumed to be 0 mL (Sagawa, 1981; Stevenson et al., 2012a, 2012b):

(6)
$$\text{LVEF}=\frac{\frac{\;P_{a}\left(n_{\mathrm{bs}}T\right)}{\;C_{a}\;E_{\mathrm{LV}}\left(n_{\mathrm{bs}}T\right)}-\frac{\;P_{a}\left(n_{\mathrm{es}}T\right)}{\;C_{a}\;E_{\mathrm{LV}}\left(n_{\mathrm{es}}T\right)}}{\frac{\;P_{a}\left(n_{\mathrm{bs}}T\right)}{\;C_{a}\;E_{\mathrm{LV}}\left(n_{\mathrm{bs}}T\right)}}\times 100\%$$
